# Undernutrition and Its Correlates among Children of 3–9 Years of Age Residing in Slum Areas of Bhubaneswar, India

**DOI:** 10.1155/2014/719673

**Published:** 2014-12-17

**Authors:** Ansuman Panigrahi, Sai Chandan Das

**Affiliations:** Department of Community Medicine, Kalinga Institute of Medical Sciences, KIIT University Bhubaneswar, Odisha 751024, India

## Abstract

Undernutrition among children is a major public health concern worldwide, more prevalent in Asia and Africa. It manifests itself in various forms such as wasting or stunting or underweight and retards physical and mental development, increases susceptibility to infection, and reduces educational attainment and productivity. The present study was undertaken to assess the level of wasting, stunting, and underweight and determine its associates among slum children of 3–9 years of age, residing in Bhubaneswar city, India. After obtaining informed consent, a total of 249 children from 249 households were studied and their parents/guardians were interviewed to collect all relevant information. 23.3%, 57.4%, and 45.4% of children were found to have wasting, stunting, and underweight, respectively. Variables like birth order of child, period of initiation of breastfeeding and mother's education were found to be strong predictors of wasting, whereas toilet facility in household and practice of drinking water storage were significantly associated with stunting among slum children as revealed in multiple regression analysis. Thus, a multipronged approach is needed such as giving priority to improve education for slum community especially for women, creating awareness regarding benefits of early initiation of breastfeeding, small family size, and proper storage of drinking water, and providing toilet facility in slum households which could improve the nutritional status of slum children.

## 1. Introduction

Nutrition has been recognized as a basic pillar for social and economic development. Adequate nutrition is necessary in early childhood to ensure healthy growth, proper functioning of organs, strong immune system, and neurological and cognitive development [1]. Undernutrition is a leading contributor to infant, child, and maternal morbidity and mortality playing a role in about half of all child deaths [1, 2]. Undernutrition directly affects many aspects of children's development such as retarding physical and mental development, increasing susceptibility to infections like sepsis, diarrhoea, pneumonia, and so forth, and further enhancing the probability of undernutrition [2, 3]. It also undermines education attainment and productivity, thereby affecting the economic growth. The prevalence of underweight children in India is among the highest in the world and is nearly double that of sub-Saharan Africa [4]. Undernutrition manifests itself in different forms in children such as wasting (indicator of acute undernutrition) or stunting (indicator of chronic undernutrition) or underweight (combined indicator for both acute and chronic undernutrition) [3].

According to WHO-UNICEF-World Bank report, it was estimated that in 2012 about 162 million, 99 million, and 51 million of under five children were stunted, underweight, and wasted, respectively, worldwide. More than half of these children lived in Asia [5]. According to the National Family Health Survey-3, among under five children, about 48%, 43%, and 20% are stunted, underweight, and wasted, respectively, whereas about 24% are severely stunted and 16% are severely underweight [6]. Odisha is one of the high prevalence states of India, where almost 50% of under five children are underweight [2, 4].

Urban slum dwellers are exposed to poor housing, overcrowding, poor quality drinking water, inadequate sanitation, which is further aggravated by their ignorance, illiteracy, and low socioeconomic status, and lack of access to basic health care facilities. Children living under such conditions are always at a high risk of developing health and nutritional problems [7]. Although numerous studies have been done in different states of India to assess the nutritional status of children, there are very limited studies involving children both in preschool age and in school age group, especially in Odisha, one of the high prevalence states of India. Thus, the present study was undertaken to ascertain the level of different forms of undernutrition such as wasting, stunting, and underweight and determine its associates among slum children of 3–9 years residing in Bhubaneswar city, Odisha.

## 2. Methodology

The present cross-sectional study was carried out in urban slums of Bhubaneswar, Odisha, India, involving children of age group (3–9 years) during the year 2013-2014. The sample size of 190 was calculated assuming the prevalence of undernutrition as 45% [8], precision of 10% at 95% confidence interval, with a design effect of 2. Multistage cluster random sampling technique was adopted to select the study population.

Bhubaneswar city has been divided into 5 geographical zones such as North, East, Central, West, and South, out of which 3 were selected randomly. From each zone, one ward was randomly chosen and then all the notified slums in the selected wards were considered for the study purpose. Overall, 6 slums were considered as study clusters. It was decided to include 40 households from each of the selected slum areas for the study purpose. In each household, only one child aged between 3 and 9 years having no known health disorder was randomly chosen as study subject. However, a total of 249 children of 3–9 years of age were included in the study. Their anthropometric measurements such as height and weight were taken by the investigators and trained field workers. A predesigned and pretested schedule was used to elicit relevant information regarding sociodemographic and individual characteristics like age, sex, education and occupation of parents, per capita monthly income, type of family, mother's age at the time of birth of the child, birth order, and exclusive breastfeeding, and environmental information such as overcrowding, toilet facility, and drinking water storage.

Ethical approval was obtained from the Institutional Ethics Committee of Kalinga Institute of Medical Sciences, Bhubaneswar. For participation of the study subjects, parents/guardians were informed about the study objectives and verbal consent was obtained prior to inclusion in the study.

Weight and height measurements were made following standard operating procedures. Weight was measured to the nearest 0.5 kg in a standard weighing (bathroom) scale (model number PL8019, NOVA BS 1120/00). Weighing scale was calibrated every time before a new measurement was taken. Children were asked to stand still over the centre of the scale with minimal clothing and without footwear and hold their head up and face forward, arms hanging freely by the sides of the body, with palms facing the thigh. Height was measured to the nearest 0.1 cm with nonstretchable tape (TR-13–60′′ Tailor's tape) which was fixed to a vertical smooth wall and the subject was asked to stand erect without footwear on a firm/level surface with his/her back against the wall, feet parallel, and hands hanging by the sides. Each measurement was done twice and the average of the two readings was recorded. Anthropometric data were analysed using WHO AnthroPlus version 1.0.4 software for assessing the growth of the children. The children were classified using the following categories.

1. Wasting (acute undernutrition) is defined as a low weight for height. Children with *z* scores (WHZ) <−2 are considered as wasted and those with WHZ <−3 are severely wasted.
2. Stunting (chronic undernutrition) is defined as a low height for age. Children with *z* scores (HAZ) <−2 are considered as stunted and those with HAZ <−3 are severely stunted.
3. Underweight (mixed acute and chronic undernutrition) is defined as a low weight for age. Children with *z* scores (WAZ) <−2 are considered as underweight and those with WAZ <−3 are severely underweight.
4. Thinness (measure of body fat) is defined as a low body mass index. Children with *z* scores (BMIZ) <−2 are considered as thin and those with BMI <−3 are severely thin.

All the data were compiled and analysed using Statistical Package for Social Sciences (SPSS) software version 16.0 and appropriate statistical tests were applied. “*P*” value <0.05 was considered as statistically significant. Univariate as well as multivariate analyses were carried out to test associations between various individual and sociodemographic characteristics and nutritional status of children. The variables found to have “*P*” value <0.25 in univariate analysis were entered into logistic regression model. As multistage sampling technique was used for data collection, sample weights were calculated and taken into consideration during the data analysis in order to get more valid results.

## 3. Results

Overall, 249 slum children of age group between 3 and 9 years were surveyed. Majority of the study subjects were girls (68.3%) and the rest were boys (31.7%). Mean age with standard deviation (SD) of girls and boys was 5.07 ± 1.6 years and 5.75 ± 2.07 years, respectively. The mean height (with SD) of the girls was 100.3 ± 11.5 cm, whereas that of boys was 103.1 ± 12.8 cm. The mean weight (with SD) of the girls was 15.0 ± 3.5 kg, whereas that of boys was 15.7 ± 4.33 kg. The anthropometric measurements such as height and weight were found to be more in boys than in girls in all the age groups.

Table 1 shows the nutritional status among slum children. Out of 249 children, 58 (23.3%), 143 (57.4%), 113 (45.4%), and 57 (22.9%) were wasted/severely wasted, stunted/severely stunted, underweight/severely underweight, and thin/very thin, respectively. Overall, 65.5% of slum children were found to have undernutrition.

Univariate analysis showing association between wasting and various individual and sociodemographic characteristics is given in Table 2. The prevalence of wasting was found to be more in children in higher age category and more boys had wasting than girls, though these differences are statistically insignificant. More children with birth order ≥2 were suffering from wasting as compared to children having first birth order, which is statistically significant (*P* = 0.010). Higher proportions of wasted children were observed in mothers having lower education and vice versa. Hence, higher maternal education appeared to be associated with better child nutrition (*P* = 0.056). Almost similar pattern of child's nutrition was found with father's education. Further, households where the practice of storing drinking water was satisfactory had less number of wasted children (13.9%) as compared to 27.6% children in households with unsatisfactory practice of drinking water storage. This difference is statistically significant (*P* = 0.016). Other factors such as period of initiation of breastfeeding, mother's age at birth of child, type of family, per capita monthly income, presence of overcrowding, and toilet facility in household were not found to be significantly associated with wasting among slum children.

All the variables found to have “*P*” value <0.25 in the univariate analysis were entered into logistic regression model (Table 3). When controlling for other variables, practice of drinking water storage lost its significance. However, birth order of child, period of initiation of breastfeeding, and mother's education came out as strong independent predictors of wasting in slum children.

Table 4 depicts the association between stunting among slum children and various individual and sociodemographic characteristics. In contrast to wasting, the prevalence of stunting was more among children of lower age. However more boys (63.3%) were found to be stunted than their counterparts (54.7%). The mother's age at child's birth appeared to be significant risk factor for stunting in children as more children (71.4%) born to younger mothers (≤20 years of age) had stunting compared to children born to older women (*P* = 0.015). Households having toilet facilities had significantly less number of stunted children (52.4%) as compared to 67.9% children in households without toilet facilities (*P* = 0.019). It was also observed that the practice of storing drinking water satisfactorily in the households had a significant positive impact on the child nutrition (*P* = 0.007) as more (63.5%) children were having stunting in households where practice of drinking water storage was unsatisfactory compared to less (44.3%) number of children in households having satisfactory storage of drinking water. Other factors such as birth order of the child, period of initiation of breastfeeding, parents' education, type of family, per capita monthly income, and overcrowding were not found to significantly affect the status of stunting among slum children.

All the variables found to have *P* < 0.25 in the univariate analysis were entered into logistic regression model (Table 5). Variables like toilet facility and practice of drinking water storage in household came out as strong predictors of stunting among slum children, whereas mother's age at birth of child lost its significance.

## 4. Discussion

Childhood undernutrition is a major public health problem in India especially in slums [9–12]. Starting with the positive aspect of results found in the present study, it was found that almost 90% of slum children were completely immunised till date, but the prevalence of undernutrition was still very high (65.5%). Taking different indicators of undernutrition into account, wasting (23.3%), stunting (57.4%), and underweight (45.4%), found in the children under study, is unacceptably high. Most studies worldwide have also reported high rates of undernutrition among slum children [8, 13–16]. The results of the present study are consistent with these findings.

Various studies have established the fact that boys are more likely to be stunted, wasted, and underweight than girls [17–20], which was also observed in the present study. In contrast, some other studies found no such association between gender and undernutrition [21–23].

Logistic regression analysis revealed the net effect of individual predictor on the dependent variable. It was observed that children of birth order of 3 or more were almost three times having wasting than children of first order. Children with higher birth order might get less attention and care compared to children of first order. Elkholy et al. revealed in their study that high means of wasting were found among the group of high birth order [24]. Association of undernutrition with high birth order has been shown by many studies [25–28]. The odds of having wasting rise 4.2 times in case of children whose mothers initiated breastfeeding after 24 hours of birth as compared to children who were breastfed within 1 hour of their birth. Early initiation of breastfeeding positively affects child's overall nutritional status [29, 30]. Mother's education was found to have a strong independent effect on child's nutrition controlling the effects of other variables. Illiterate mothers and mothers with primary education were about thrice more likely to have wasted children as compared to mothers who had attained higher education (high school and above). It might be due to the reason that higher educated women can take independent decisions and have greater exposure to outside world and thus access to various resources which help them in securing proper nutrition of their children. They are more aware about proper nutrition, maintenance of hygiene, and various health issues as compared to uneducated or less educated women. Earlier studies have also documented the positive impact of mother's education on wasting [31–33].

Two independent predictors of stunting were also identified. Children residing in households without having toilet facility were 1.5 times more at risk of developing stunting as compared to children in households with toilet facility. This finding has been corroborated in other studies [34–37]. Unavailability of toilet facility in households would promote open defecation which facilitates various water borne diseases that can negatively affect the health and nutrition of young children. Further, children in households with unsatisfactory practice of storing drinking water were 2.2 times more likely to have stunting than children living in households with satisfactory practice of drinking water storage. Merchant et al. showed in their study that children coming from homes with water and sanitation had a 17% greater chance of reversing stunting than those coming from homes without either facility [35]. Similar results have also been found in other studies [38, 39].

The estimation in the study is not a good measure of causal association between independent variables and nutrition of children because of cross-sectional nature of the study. There might be recall bias as some of the information was based on response of the study participants. In spite of these limitations, the study has certain important implications.

## 5. Conclusion and Recommendation

The present study was probably the first of its kind in Odisha to assess the nutritional status among the slum children both in preschool age and in primary school age group using WHO child growth standards. It is evident from the study that nutritional status of children shows a gloomy picture in slums of Odisha. Thus, utmost care and attention must be focused on these socioeconomically and disadvantaged children living in slums to attain the MDG goals. Sincere efforts must be undertaken to make a significant impact on child's nutrition with multipronged approach such as giving priority to education for slum community especially for women, creating awareness regarding benefits of factors like early initiation of breastfeeding, limiting family size, proper storage of drinking water, and so forth, and providing toilet facility in the household. Similar studies need to be carried out among slum children of different regions to find out whether there are any ethnic and regional variations in the prevalence of undernutrition and also operational research can be planned regarding deliverance of multipronged strategies to curb this public health menace.

## Figures and Tables

**Table 1:** Nutritional status of children (3–9 years) in urban slums of Bhubaneswar, India (*n* = 249).

Indicator	Number	Percentage
Weight for height		
Normal	191	76.7
Wasted	44	17.7
Severely wasted	14	5.6
Height for age		
Normal	106	42.6
Stunted	64	25.7
Severely stunted	79	31.7
Weight for age		
Normal	136	54.6
Underweight	42	16.9
Severely underweight	71	28.5
Body mass index		
Normal	192	77.1
Thin	42	16.9
Very thin	15	6.0

**Table 2:** Univariate model showing association between wasting among slum children and various individual and sociodemographic characteristics.

Independent variable	Wasting		Total (%)	“*P*” value^*^
	Absent	Present		
	Number (%)	Number (%)		
Age (in years) of child				
3–6	132 (82.5)	28 (17.5)	160 (64.3)	0.118
6–9	59 (66.3)	30 (33.7)	89 (35.7)	
Gender of child				
Female	135 (79.4)	35 (20.6)	170 (68.3)	0.117
Male	56 (70.9)	23 (29.1)	79 (31.7)	
Birth order of child				
First	74 (89.2)	09 (10.8)	83 (33.3)	**0.010**
Second	87 (70.2)	37 (29.8)	124 (49.8)	
Third	30 (71.4)	12 (28.6)	42 (16.9)	
Initiation of breastfeeding				
Within 1 hr	89 (82.4)	19 (17.6)	108 (43.4)	0.083
Between 1 and 24 hrs	83 (75.5)	27 (24.5)	110 (44.2)	
After 24 hrs	19 (61.3)	12 (38.7)	31 (12.4)	
Mother's age (in years) at birth of child				
≤20	57 (81.4)	13 (18.6)	70 (28.1)	0.231
21–30	108 (74)	38 (26)	146 (58.6)	
≥30	26 (78.8)	07 (21.2)	33 (13.3)	
Mother's education				
Illiterate	48 (68.6)	22 (31.4)	70 (28.1)	0.056
Primary	54 (73.0)	20 (27.0)	74 (29.7)	
Middle	63 (82.9)	13 (17.1)	76 (30.5)	
High school and above	26 (89.7)	03 (10.3)	29 (11.7)	
Father's education				
Illiterate	51 (67.1)	25 (32.9)	76 (30.5)	0.071
Primary	45 (70.3)	19 (29.7)	64 (25.7)	
Middle	58 (87.9)	08 (12.1)	66 (26.5)	
High school and above	37 (86.0)	06 (14.0)	43 (17.3)	
Type of family^a^				
Joint	25 (80.6)	06 (19.4)	31 (12.4)	0.573
Nuclear	166 (76.1)	52 (23.9)	218 (87.6)	
Per capita monthly income^b^				
<Rs. 2000	29 (65.9)	15 (34.1)	44 (17.7)	0.262
Rs. 2000–Rs. 4000	104 (78.2)	29 (21.8)	133 (53.4)	
≥Rs. 4000	56 (80.6)	14 (19.4)	72 (28.9)	
Overcrowding				
Absent	58 (80.6)	14 (19.4)	72 (28.9)	0.072
Present	133 (75.1)	44 (24.9)	177 (71.1)	
Toilet facility				
Absent	61 (75.3)	20 (24.7)	81 (32.5)	0.694
Present	130 (77.4)	38 (22.6)	168 (67.5)	
Drinking water storage				
Satisfactory	68 (86.1)	11 (13.9)	79 (31.7)	**0.016**
Unsatisfactory	123 (72.4)	47 (27.6)	170 (68.3)	

^*^Significance is based on adjusted chi-square statistic.

^a^Joint family consists of two or more married couples and their children living together in the same household, all the men being related by blood of patrilineal descent. Nuclear family consists of the married couple and their dependent children, residing in the same household.

^b^One US dollar is equivalent to Rs. 60 (approximately).

**Table 3:** Multiple logistic regression model showing association between wasting among slum children and various factors.

Independent variable	Dependent variable (wasting)		*P* value^*^
	Normal = reference category		
	*β*	Exp(*β*)	
Age (in years) of child			
3–6	−0.762	0.467	0.220
6–9^R^			
Gender of child			
Female	−0.480	0.619	0.053
Male^R^			
Birth order of child			
First	−1.059	0.347	**0.013**
Second	0.378	1.460	0.392
Third or more^R^			
Initiation of breastfeeding			
Within 1 hr	−1.445	0.236	**0.035**
Between 1 and 24 hrs	−0.713	0.490	0.151
After 24 hrs^R^			
Mother's age (in years) at birth of child			
≤20	0.190	1.209	0.522
21–30	0.174	1.190	0.534
≥30^R^			
Mother's education			
Illiterate	1.179	3.250	**0.024**
Primary	1.136	3.115	**0.024**
Middle	0.558	1.747	0.329
High school and above^R^			
Father's education			
Illiterate	1.036	2.817	0.208
Primary	0.835	2.304	0.303
Middle	−0.114	0.892	0.792
High school and above^R^			
Overcrowding			
Absent	−0.499	0.607	0.147
Present^R^			
Drinking water storage			
Satisfactory	−0.909	0.403	0.099
Unsatisfactory^R^			

Note: R = reference category, *β* = regression coefficient (log odds ratio), and Exp(*β*) = odds ratio.

Model fit statistics: pseudo-*R* squares—Cox and Snell = 0.188, Nagelkerke = 0.283, and Mcfadden = 0.191; classification table reports that the overall expected model performance is 81.1%; that is, 81.1% of the cases can be expected to be classified correctly by the model.

**Table 4:** Univariate model showing association between stunting among slum children and various individual and sociodemographic characteristics.

Independent variable	Stunting		Total (%)	“*P*” value^*^
	Absent	Present		
	Number (%)	Number (%)		
Age (in years) of child				
3–6	63 (39.4)	97 (60.6)	160 (64.3)	0.190
6–9	43 (48.3)	46 (51.7)	89 (35.7)	
Gender of child				
Female	77 (45.2)	93 (54.7)	170 (68.3)	0.058
Male	29 (36.7)	50 (63.3)	79 (31.7)	
Birth order of child				
First	33 (39.8)	50 (60.2)	83 (33.3)	0.429
Second	54 (43.5)	70 (56.5)	124 (49.8)	
Third	19 (45.2)	23 (54.8)	42 (16.9)	
Initiation of breastfeeding				
Within 1 hr	44 (40.7)	64 (59.3)	108 (43.4)	0.528
Between 1 and 24 hrs	50 (45.5)	60 (54.5)	110 (44.2)	
After 24 hrs	12 (38.7)	19 (61.3)	31 (12.4)	
Mother's age (in years) at birth of child				
≤20	20 (28.6)	50 (71.4)	70 (28.1)	**0.015**
21–30	72 (49.3)	74 (50.7)	146 (58.6)	
≥30	14 (42.4)	19 (57.6)	33 (13.3)	
Mother's education				
Illiterate	23 (32.9)	47 (67.1)	70 (28.1)	0.093
Primary	24 (32.4)	50 (67.6)	74 (29.7)	
Middle	39 (51.3)	37 (48.7)	76 (30.5)	
High school and above	20 (69.0)	09 (31.0)	29 (11.7)	
Father's education				
Illiterate	21 (27.6)	55 (72.4)	76 (30.5)	0.107
Primary	29 (45.3)	35 (54.7)	64 (25.7)	
Middle	32 (48.5)	34 (51.5)	66 (26.5)	
High school and above	24 (55.8)	19 (44.2)	43 (17.3)	
Type of family^a^				
Joint	10 (32.3)	21 (67.7)	31 (12.4)	0.327
Nuclear	96 (44)	122 (56.0)	218 (87.6)	
Per capita monthly income^b^				
<Rs. 2000	14 (31.8)	30 (68.2)	44 (17.7)	0.235
Rs. 2000–Rs. 4000	53 (39.8)	80 (60.2)	133 (53.4)	
≥Rs. 4000	39 (54.2)	33 (45.8)	72 (28.9)	
Overcrowding				
Absent	34 (47.2)	38 (52.8)	72 (28.9)	0.387
Present	72 (40.7)	105 (59.3)	177 (71.1)	
Toilet facility				
Absent	26 (32.1)	55 (67.9)	81 (32.5)	**0.019**
Present	80 (47.6)	88 (52.4)	168 (67.5)	
Drinking water storage				
Satisfactory	44 (55.7)	35 (44.3)	79 (31.7)	**0.007**
Unsatisfactory	62 (36.5)	108 (63.5)	170 (68.3)	

^*^Significance is based on adjusted chi-square statistic.

^a^Joint family consists of two or more married couples and their children living together in the same household, all the men being related by blood of patrilineal descent. Nuclear family consists of the married couple and their dependent children, residing in the same household.

^b^One US dollar is equivalent to Rs. 60 (approximately).

**Table 5:** Multiple logistic regression model showing association between stunting among slum children and various factors.

Independent variable	Dependent variable (wasting)		*P* value^*^
	Normal = reference category		
	*β*	Exp(*β*)	
Age (in years) of child			
3–6	0.334	1.396	0.207
6–9^R^			
Gender of child			
Female	−0.360	0.698	0.076
Male^R^			
Mother's age (in years) at birth of child			
≤20	0.371	1.450	0.187
21–30	−0.471	0.624	0.173
≥30^R^			
Mother's education			
Illiterate	1.023	2.783	0.146
Primary	1.056	2.875	0.133
Middle	0.370	1.447	0.605
High school and above^R^			
Father's education			
Illiterate	0.984	2.674	0.134
Primary	0.201	1.223	0.688
Middle	−0.042	1.043	0.919
High school and above^R^			
Per capita monthly income			
<Rs. 2000	1.003	2.725	0.100
Rs. 2000–Rs. 4000	0.634	1.885	0.190
≥Rs. 4000^R^			
Toilet facility			
Absent	0.436	1.546	**0.031**
Present			
Drinking water storage			
Satisfactory	−0.822	0.439	**0.047**
Unsatisfactory^R^			

Note: R = reference category, *β* = regression coefficient (log odds ratio), and Exp(*β*) = odds ratio.

Model fit statistics: pseudo-*R* squares—Cox and Snell = 0.180, Nagelkerke = 0.241, and Mcfadden = 0.145; classification table reports that the overall expected model performance is 71.1%; that is, 71.1% of the cases can be expected to be classified correctly by the model.
